# Transcranial Ultrasound Stimulation of the Anterior Cingulate Cortex Reduces Neuropathic Pain in Mice

**DOI:** 10.1155/2021/6510383

**Published:** 2021-12-31

**Authors:** Xiangjun Feng, Lili Niu, Meng Long, Kaixuan Luo, Xiaowei Huang, Moxian Chen, Zhengrong Lin, Wei Zhou, Shasha Yi, Lijuan Ao

**Affiliations:** ^1^The School of Rehabilitation, Kunming Medical University, 1168 West Chunrong Road, Chenggong, Kunming 650500, China; ^2^General Surgery Department of Geriatrics, The First Affiliated Hospital of Kunming Medical University, 295 Xichang Road, Kunming 650032, China; ^3^Institute of Biomedical and Health Engineering, Shenzhen Institutes of Advanced Technology, Chinese Academy of Sciences, 1068 Xueyuan Avenue, Shenzhen 518055, China; ^4^CAS Key Laboratory of Health Informatics, Shenzhen Institutes of Advanced Technology, 1068 Xueyuan Avenue, Shenzhen 518055, China; ^5^Guangdong-Hong Kong-Macao Greater Bay Area Center for Brain Science and Brain-Inspired Intelligence, 1068 Xueyuan Avenue, Guangzhou 518055, China

## Abstract

Focused ultrasound (FUS) is a potential tool for treating chronic pain by modulating the central nervous system. Herein, we aimed to determine whether transcranial FUS stimulation of the anterior cingulate cortex (ACC) effectively improved chronic pain in the chronic compress injury mice model at different stages of neuropathic pain. The mechanical threshold of pain was recorded in the nociceptive tests. We found FUS stimulation elevated the mechanical threshold of pain in both short-term (*p* < 0.01) and long-term (*p* < 0.05) experiments. Furthermore, we determined protein expression differences in ACC between the control group, the intervention group, and the Sham group to analyze the underlying mechanism of FUS stimulation in improving neuropathic pain. Additionally, the results showed FUS stimulation led to alterations in differential proteins in long-term experiments, including cellular processes, cellular signaling, and information storage and processing. Our findings indicate FUS may effectively alleviate mechanical neuropathic pain via the ACC's stimulation, especially in the chronic state.

## 1. Introduction

Chronic pain is a severe condition that considerably interferes with daily functioning [1], and the estimated prevalence of chronic pain ranges from 8.7% to 64.4% [2, 3]. Neuropathic pain (NP) is a prominent type of chronic pain. Chronic pain causes not only stress on the body, including strained muscles, diminished motion range, inadequate power, and appetite changes, but also emotional effects, such as depression, anger, anxiety, and fear of reinjury, which may limit the ability to return to routine work or leisure activities [4]. The treatment is mainly medication or physical therapy, but the effect is not significant due to the complicated etiology [5, 6]. Therefore, it is necessary to develop effective strategies to improve these issues.

The use of the S-size ultrasound probe in pulsed mode stimulation over a skin incision has been reported to improve the mechanical and thermal retraction threshold of the NP model [7]. A previous study reported that axonal regeneration in autograft nerves was improved following low-intensity pulsed ultrasound with 250 mW/cm^2^ compared with that following high-intensity ultrasound [8]. Therefore, focused ultrasound (FUS) may be a nonpharmacological nonablative neuromodulatory technique that improves peripheral nerve injury or NP. Additionally, Hameroff et al. stimulated individuals with chronic pain using transcranial ultrasound and reported an unexpected analgesic effect [9]. Moreover, Spooner et al. reported that deep brain stimulation over the bilateral cingulate by 130 Hz high-frequency electricity resulted in pain relief in a patient with severe drug-resistant pain syndrome following a complete spinal cord injury [10]. Subsequently, there has been increasing interest in modulating the central nervous system (CNS) for chronic pain treatment.

Thalamus is an important regulatory target for the treatment of pain, and ACC is a possible target for pain management through previous researches. Pain relief using cingulotomy has evoked clinical interest in deep brain stimulation in the dorsal ACC for treating chronic refractory pain, especially when coupled with a substantial affective component, such as distress, resulting in the more complicated treatment [11]. ACC activation improves chronic pain states through several neuronal modulation changes in the CNS [12–14]. Moon et al. reported that optical inhibition of the ACC improved pain-associated behavior and reduced the unusual activity of thalamic sensory neurons in a rat model of trigeminal NP [15]. In addition, various neuromodulation techniques have demonstrated therapeutic value against NP by inhibiting neuronal activity in the ACC, a crucial target in the brain [13, 15–17].

At present, deep brain stimulation, transcranial magnetic stimulation, and transcranial electrical stimulation are the most commonly used regulation programs of the CNS, which may have the disadvantages of high surgical risk and poor accuracy. Recent studies have also shown that FUS is a safe, noninvasive, and accurate technique that modulates neuronal circuits in the CNS [18, 19] of both animal models and humans [20–22]. Moreover, transcranial FUS may treat chronic pain through neuronal regulation of the CNS, including the ACC. However, the treatment effect of FUS-induced ACC activation on chronic pain remains unclear. Consequently, we investigated the therapeutic effects of FUS stimulation of the ACC using the short- and long-term NP chronic compress injury (CCI) mouse model.

## 2. Materials and Methods

### 2.1. Animal Preparation

We conducted all animal experiments based on the guidelines approved by the Animal Use Committee and the Ethics Committee of Kunming Medical University (Approval number: KMMU2019075). For short-term experiments, we randomly allocated 18 C57BL/6J mice (age: 8 weeks, weight: 20–23 g, male) to the FUS1 (parameter 1), FUS2 (parameter 2), and control (sham stimulation) groups (*n* = 6 mice/group). For the long-term experiments, we randomly allocated 36 C57BL/6J mice (age: 14 months, weight: 24–36 g, female) to the Control (Sham operation), FUS (parameter 2), and Sham (sham stimulation) groups (*n* = 12 mice/group). All animals were raised in a controlled environment (22 ± 2°C) under a regular light-dark cycle (lights on, 7 a.m.; lights off, 7 p.m.) with ad libitum access to food and water.

### 2.2. Chronic Pain Model

Following a 1-week acclimation of the mice to the maintenance environment, we began the experiment. We used the CCI surgical procedure to establish the NP model [23]. We conducted nociceptive tests, including the mechanical allodynia test, thermal allodynia test, and sciatic nerve functional index, to obtain baseline values before the CCI surgical procedure on the right sciatic nerve. The mice were anesthetized with isoflurane (2% for induction and 1.5% for maintenance, Sigma-Aldrich, St. Louis, Missouri, USA) and placed in the prone position. Next, the right thigh was shaved, and the skin was disinfected with 2% iodine-alcohol. After a skin incision was made in the middle third of the right hind limb to expose the biceps femoris muscle, approximately 5 mm of the sciatic nerve was uncovered, and three ligatures (gut ligatures 6.0, Jinhuang, Shanghai, China) were tied at 1-mm intervals. The tying allowed noticeable nerve constriction without arresting epineurial blood flow. The skin was sutured using Mononylon 4.0 (Johnson & Johnson Medical N.V., Belgium, 2018–2019). Mice in the sham-operated group were operated according to the abovementioned surgical procedure, but after the nerve was exposed, an intestinal ligature was placed on the sciatic nerve trunk three times without ligation; the nerve was repositioned, and the skin was sutured We returned the animals to their cages after surgery and recovery from anesthesia.

### 2.3. Nociceptive Tests

We conducted nociceptive tests before (baseline) and following surgery in all experimental groups. In the short-term experiment, we started ultrasound stimulation when there was a substantial decrease in the mechanical pain threshold of the entire group (≈0.008 g; ≈6 days following surgery). A substantial reduction in the mechanical pain threshold during the postoperative examination indicated successful establishment of the pain model in the long-term experiment. We started ultrasound stimulation on the 91^st^ days after the surgery and repeated the nociceptive tests every 6–7 days following ultrasound stimulation commencement.

#### 2.3.1. Mechanical Allodynia Test

All the animals were allowed 2–3 days to acclimate to the test environment before the tests or surgery. We placed the mice in a ten-grid Plexiglas box (homemade) with a metal net lid and bottom for about 30 min. Next, we perpendicularly stimulated the bilateral hind paws on the plantar surface in an ascending order of stimulus intensity using Von Frey hairs (Aesthesio, DanMic Global, Campbell, CA) with increasing stiffness (0.008, 0.02, 0.04, 0.07, 0.16, 0.4, 0.6, 1, 1.4, 2, and 4 g). A positive situation was defined when the mice ran away or raised the hind leg following a 5–6 s perpendicular stimulation. We recorded the gradually growing stiffness when a positive result occurred three times in five stimulations separated by more than 3 min. We repeated the process mentioned above 3–5 times at intervals of >15 min and obtained the mean value as the mechanical withdrawal threshold to indicate the mechanical pain tolerance of mice.

#### 2.3.2. Thermal Allodynia Test

We used a laser transmitter (ADR-1805, Xilongguangdian, Shanghai, China) to determine the thermal allodynia value with a near-infrared laser (wavelength, 787.7 nm; output power, 564 mW) that was calibrated using an infrared thermal imager (R300, NEC Avio, Tokyo, Japan). The calibration warmed the paw sole skin to 50°C at a distance of 0.5 cm within 28 s. The mice were allowed to habituate to the Plexiglas box for at least 30 min. The light was transmitted to the paw sole skin using the laser transmitter at a distance of approximately 0.5 cm. The time from the start of lighting to paw withdrawal was recorded with a 60-s limit to avoid local burn injury. This described procedure was performed twice at a 15-min interval, and the mean value was used as the thermal withdrawal threshold to indicate the thermal pain tolerance of mice.

#### 2.3.3. Sciatic Nerve Functional Index (SFI)

We prepared a plastic corridor (5 cm × 10 cm × 15 cm) with paper tape (6 cm × 20 cm, homemade) at the bottom and an ink pad at the beginning of the corridor for the mice to walk through. The mice were briefly placed on the ink pad and walked straight through the corridor while leaving footprints on the paper tape prior to and following the CCI surgery. When there were three clear footprints, we measured the following three parameters: print length (PL), distance from the heel to the third toe; toe spread (TS), distance from the first toe to the fifth toe; and intermediate TS (ITS), distance from the second toe to the fourth toe. We calculated the SFI using the following formula [24]:

(1) $$\begin{array}{c} \text{SFI}=38.3\left(\frac{\text{EPL}-\text{NPL}}{\text{NPL}}\right)+109.5\left(\frac{\text{ETS}-\text{NTS}}{\text{NTS}}\right)+13.3\left(\frac{\text{EITS}-\text{NITS}}{\text{NITS}}\right)-8.8, \end{array}$$

where *E* represents the experimental side and *N* represents the normal side. A value of 0 indicates the normal function, and −100 indicates total impairment.

### 2.4. FUS Stimulation Process

**Figure 1:**
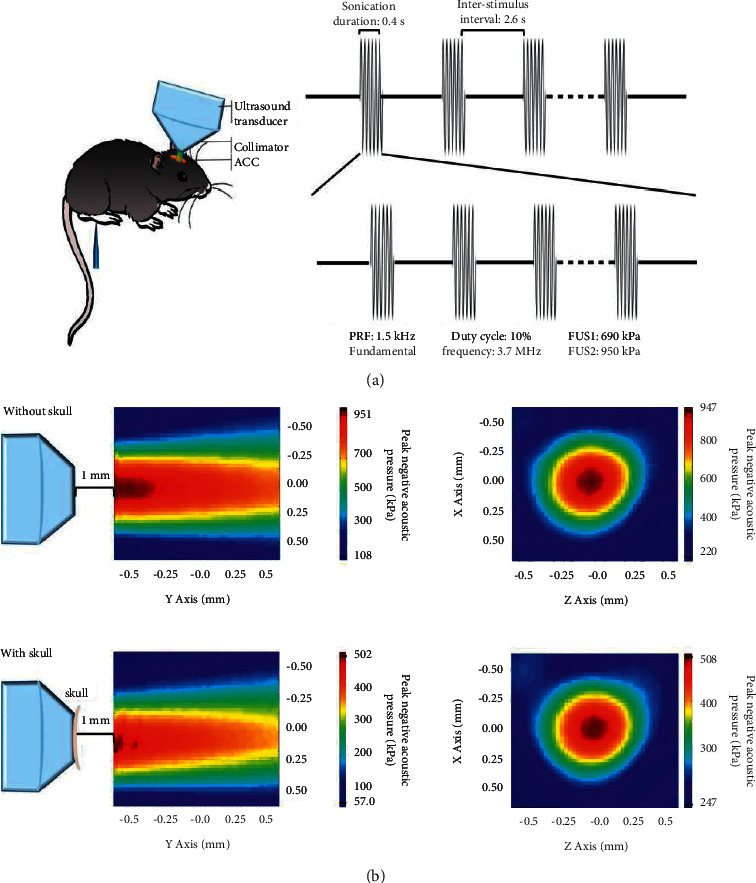
Experimental schematics. Experimental schematic diagram (a). Acoustic field distributions with or without a fresh mouse skull (b).

We used an arbitrary waveform generator (3102C, Tektronix, Texas, USA) with a power amplifier (LZY-22+, Mini-circuits, Shenzhen, China) to provide an output to apply to a 3.7 MHz focused transducer with a focal diameter and length of 0.7 and 11.1 mm, respectively. We performed FUS stimulation of the ACC (Cg) region of mice to induce pain relief in the mice (the ultrasound pulse parameters were as follows: pulse repetition frequency, 1.5 kHz and duty cycle, 10%. The spatial-peak pulse-averaged acoustic intensity (Isppa) was 15980 mW/cm^2^ and 34982 mW/cm^2^ for FUS1 and FUS2, respectively. Moreover, the spatial-peak temporal-average intensity (Ispta) was 1598 mW/cm^2^ and 3498 mW/cm^2^ for FUS1 and FUS2, respectively (Figure 1(a)). These values were obtained using degassed water through an ultrasound test tank system (Precision Acoustics Ltd., Dorchester, United Kingdom) equipped with a calibrated hydrophone (2010, Precision Acoustics Ltd., Dorchester, United Kingdom). We measured the acoustic intensity attenuation through the skull up to 80%, and the focal spot can be located just below the skull. The measured acoustic field distribution shows the measured acoustic pressure of the probe, with or without a fresh mouse skull. The focus distance for projecting across the cingulate cortex area through the collimator filled with degassed water was 11.1 mm (Figure 1(b)).

All the mice were depilated using a depilatory paste (Veet, Reckitt Benckiser, Hubei, China), and we used acoustic coupling gel for FUS treatment (15 min/day for 21 days). The mice underwent ultrasonic stimulation under anesthesia with isoflurane (2% for induction, 0.6% for maintenance, Sigma-Aldrich, St. Louis, Missouri, USA). The mice were depilated and anesthetized; then, the head of the mice was fixed on the mouse-adapted stereotaxic device. The collimator prepared by calculating the focal length of the ultrasound transducer was placed directly above the brain area of ACC (bregma point) and was coupled with a coupling agent to ensure no air was left (Figure 1(a)). Ultrasound stimulation was performed through the collimator, just touching the scalp of the mice using the above parameters, and the sham procedure was performed at the same time, which involved the same procedure of all working processes for equal time, with all equipment turned on, without ultrasonic excitation signal.

**Figure 2:**
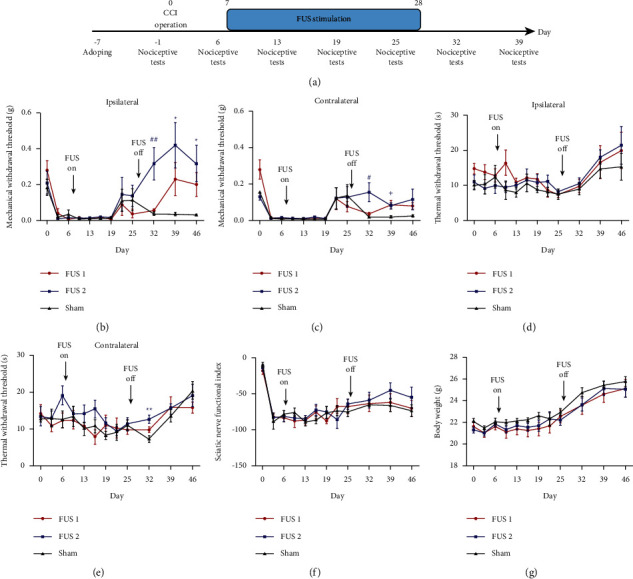
Transcranial FUS stimulation slowly improved the mechanical withdraw threshold in the short-term experiment. Timeline of the short-term experiment (*n* = 6) (a). Ultrasound stimulation increased the mechanical withdrawal thresholds of the surgical side (^##^*p*=0.003) (b). The increase in the mechanical and thermal withdrawal thresholds of the contralateral side (c, e). The variation of sciatic nerve index and body weight (f, g). ^#^ represents a statistically significant difference between the FUS2 group and the other two groups. ^*∗*^ indicates a statistically significant difference between the FUS2 group and the Sham group.  + indicates a statistically significant difference between the FUS1 group and the Sham group.

**Figure 3:**
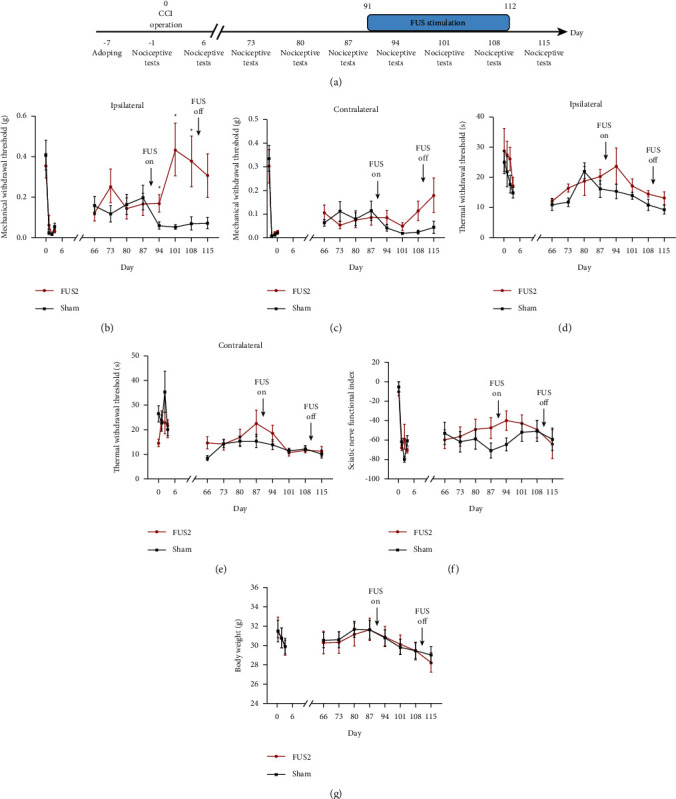
FUS stimulation promptly improved the mechanical withdraw threshold in the long-term experiment. Timeline of the long-term experiment (*n* = 12) (a). Ultrasound stimulation increased the mechanical withdraw threshold of the surgical side (*p* < 0.05) (b). There were no significant changes in the mechanical withdraw threshold of the contralateral side. The bilateral thermal withdraw threshold, body weight, and sciatic nerve index between the FUS2 group and the Sham group (c–f). ^*∗*^ indicates statistical significance between the FUS2 group and the Sham group.

First, we performed FUS on the short-term pain model to determine the effect of FUS on the ACC brain area in the early stage of pain. On the 6th day after surgery, we confirmed the successful establishment of the CCI model using the nociceptive tests. On the 7th day, we started FUS stimulation at 15 min/day for 21 days (Figure 2(a)). After completing the short-term experiment, we confirmed that FUS has a better neuromodulation effect when using the FUS2 parameters. Therefore, we conducted long-term experiments to confirm the effectiveness of the ACC regulation with FUS in the chronic pain period using FUS2 parameters. Following successful establishment of the CCI model, the mice were allowed to move freely for 90 days without intervention, and the baseline values of nociceptive tests were determined prior to ultrasound stimulation. On the 91st day, we started the FUS2 stimulation using a similar protocol to that used for the short-term experiment (Figure 3(a)).

### 2.5. Slice Preparation and Multielectrode Array (MEA) Recordings

To verify the direct effect of ultrasound on neurons in the ACC brain area using FUS2 parameters, we separately recorded three brain slices of three C57BL/6J mice for MEA recordings. The animals were sacrificed using a rodent guillotine (RWD, Shenzhen, China) under deep anesthesia with 5% isoflurane. Next, we collected the brains and placed them in an ice-cold oxygenated high-sucrose cutting solution (0–2°C) that contained the following ingredients (in mM): 60 NaCl, 3 KCl, 7 MgCl_2_, 1.25 NaH_2_PO_4_, 25 NaHCO_3_, 10 D-glucose, 115 sucrose, and 0.5 CaCl_2_. Subsequently, we prepared 500 *μ*m coronal slices of the ACC area using a vibratome (VT-1200 Series, Leica Biosystems, Wetzlar, Germany). The slices were then equilibrated and incubated in artificial cerebrospinal fluid (ACSF) that contained the following ingredients (in mM): 126 NaCl, 2.5 KCl, 1 MgCl_2_, 1.25 NaH_2_PO_4_, 26 NaHCO_3_, 10 D-glucose, 2 sodium pyruvate, 0.5 L-ascorbic acid, and 2 CaCl_2_. The ACSF was continuously saturated with 95% O_2_-5% CO_2_ and maintained at a temperature of 35°C (all the above regents: Sigma-Aldrich, St. Louis, Missouri, USA).

**Figure 4:**
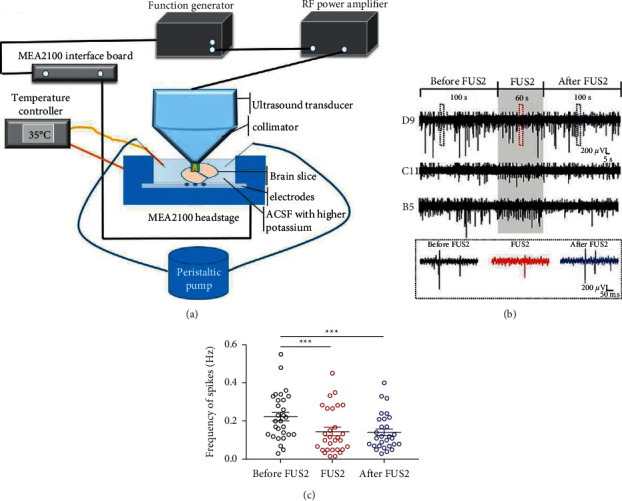
Ultrasound-induced inhibition of neuronal discharges from the ACC slices. Experimental schematics (a). Representative traces randomly selected of the neuronal discharges from the ACC slices obtained using MEA recording (b). Ultrasound stimulation substantially decreased the spike frequency of neuronal discharges (c) (^*∗∗∗*^*p* < 0.001 (prior to during: *p* ≤ 0.001, prior to following: *p*=0.002)).

To investigate the FUS-induced modulatory effects, we recorded spikes from the slices using the MEA systems (MCS MEA 2100-IFB, MCS, Reutlingen, Germany), which reliably quantifies neuronal activity [25, 26]. The brain slices were placed in the recording chamber and continuously perfused with ACSF saturated with 95% O_2_-5% CO_2_ (Figure 4(a)). We applied a solution with higher potassium levels (2.5 mM to 5 mM of KCl in the ACSF) to evoke more discharge from the brain slices. We placed an ultrasound transducer with a coupling cone (homemade) over the ACC slices and adjusted its location to ensure the acoustic field was focused on the stimulation site of the slices. We recorded the baseline value at 100 s before the ultrasound intervention. Following the record of 60-s intervention (parameter 2), we subsequently recorded the measurement value of another 100 s without intervention and identified effective neuronal discharge as those with amplitude 2.5 times greater than the baseline amplitude. There were a total of 29 channels of electrical signals recorded through MEA, indicating that the excitability of 29 neuron cells was recorded. The effective discharge times were counted manually for statistical analysis.

### 2.6. Detection of Proteins

To preliminarily determine the local protein expression changes of ACC after the neuroregulation of FUS and to understand the possible central control mechanism of pain, we conducted protein detection on the ACC brain area of the long-term experimental group using an Isobaric tag for relative and absolute quantitation (iTRAQ), which is among the most commonly used methods in quantitative proteomics research. It works on the principle of the reaction of digested polypeptides from different samples with differentially labeled iTRAQ reagents [27]. On the 21st day of the long-term experiment, 12 mice (*n* = 6/group) were cardiacally perfused with a saline solution. Subsequently, the whole brains were quickly removed, and the entire ACC tissue was obtained according to the map [28]. Following multiple sample protein extractions, we performed protein digestion and quantification based on the filter-aided sample preparation method [29]. Further, we vacuum-dried (Savant DNA120, Thermo Scientific, Massachusetts, USA) three samples with the greatest protein concentration in each group for iTRAQ-based identification. We used information-dependent acquisition (IDA) mass spectrum techniques to obtain tandem mass spectrometry (MS) data on a ThermoFisher *Q* Exactive mass spectrometer (Thermo Scientific, Massachusetts, USA) fitted with a Nano Flex ion source (Thermo Scientific, Massachusetts, USA). We performed the FULL-MS scans with an ion spray voltage of 1.9 kV and an interface heater temperature of 275°C. We obtained survey scans of IDA within 250 ms and a maximum of 20 production scans within 50 ms. With a dynamic exclusion of 25 s, fragmentation was performed with higher-energy collision energy dissociation for 2+ to 4+ charged spectra. We analyzed the MS/MS data for protein identiﬁcation and quantiﬁcation using IPeak.

### 2.7. Western Blotting

Total protein was extracted from tissues. Antibodies Hnrnph1, Snrpb, Dhx16, and GAPDH were purchased from Abcam (USA). Antibody Hnrnpd was purchased from CST (USA). Membranes were blocked with 5% milk and incubated with the primary antibodies in 5% bovine serum albumin (BSA) at 4°C overnight. Next day, after washing membrane three times, then it was incubated with 1 : 1000 secondary antibody. Then the signals were detected using an enhanced chemiluminescence-detecting kit (Thermo Fisher, MA, USA), and the density of the bands was analyzed by using ImageJ.

### 2.8. Safety and Temperature Calculations

Finally, we conducted a safety test. We performed standard hematoxylin-eosin (HE, Sigma-Aldrich, St. Louis, Missouri, USA) staining of the brain and the CCI surgical area after the experiment in all test groups to determine that FUS would not cause further tissue damage and used mathematical modeling to determine the local temperature increase. Specifically, 12 FUS2-stimulated and Sham mice (*n* = 6/group) were used for histological assessment. On the 21st day, 12 mice were cardiacally perfused with phosphate-buffered saline followed by 4% paraformaldehyde. The whole brains were then isolated and fixed in 4% paraformaldehyde (Sigma-Aldrich, St. Louis, Missouri, USA). The brain tissues were dehydrated, defatted, paraffinized, and serially sectioned at 4-*μ*m thicknesses using a pathologic microtome (Leica, RM2016, Wetzlar, Germany). We randomly selected the brain sections for observation under light microscopy. Upon the spread of ultrasonic energy into tissues, wave energy attenuation resulted from absorption or scattering with heat conversion or directional change, respectively. The balance between the absorbed energy and heat released results in an ultrasound-induced temperature increase that can be calculated using mathematical modeling techniques under different exposure conditions. Therefore, we calculated the ultrasound-induced local temperature increase to avoid local thermal damage using the following equation:

(2) $$\begin{array}{c} Q=2\alpha I_{\text{TA}}, \end{array}$$

where *α* is the absorption coefficient in the brain tissue, *I*_TA_ is the temporal-average intensity, and *Q* is the heat generated per volume [30].

The FUS-induced maximum temperature increase (Δ*T*_max_) in the brain tissue could be described as follows without the heat loss:

(3) $$\begin{array}{c} \Delta T_{\max}=\frac{Q\Delta_{t}}{c_{\upsilon }}=\frac{Q\Delta_{t}}{c_{\rho }}, \end{array}$$

where Δ_*t*_ is the FUS exposure time and *C*_*ν*_ is the heat capacity per unit volume for brain tissue defined as the product of C (heat capacity in brain tissue: 3.6 J/g/°C) and *ρ* (density of brain tissue: 1028 kg/m^3^) [31].

### 2.9. Statistical Analysis

We expressed all experimental data as mean ± standard error (SME) and conducted analyses using independent sample *t*-tests and one-way ANOVA tests with Tukey's or Bonferroni's post hoc test for parametric analysis. All statistical analyses were performed in IBM® SPSS® Statistics 23 (IBM Corp, Armonk, NY, USA), and the statistical significance was set at *p* < 0.05.

## 3. Results

### 3.1. Transcranial FUS Stimulation Slowly Improved the Mechanical Withdraw Threshold in the Short-Term Experiment

The results showed an increase in the mechanical withdraw threshold of the operation side following the end of the FUS2 stimulation period (FUS1 group, 0.06 ± 0.01 g; FUS2 group, 0.32 ± 0.09 g; Sham group, 0.04 ± 0.01 g; *p* < 0.01 (*p*=0.003)), indicating that the pain tolerance of mice on the surgical side had improved. FUS1 showed a similar but delayed and nonsignificant response. On the contralateral side, FUS2 exerted a similar effect; however, the outcome was not sustained (FUS1 group, 0.04 ± 0.01 g; FUS2 group, 0.15 ± 0.05 g; Sham group, 0.02 ± 0.002 g; *p* < 0.05 (*p*=0.038) (Figure 2(b)). Conversely, FUS1 stimulation induced a significant increase after 1 week (FUS1 group, 0.09 ± 0.02 g; FUS2 group, 0.08 ± 0.02 g; Sham group, 0.02 ± 0.005 g; *p* < 0.05 (*p*=0.038) (Figure 2(c)). There was substantial synchrony in the bilateral mechanical retraction domain following ultrasound stimulation and a significant improvement in the contralateral thermal withdrawal threshold following FUS2 stimulation (FUS1 group, 9.83 ± 0.74 s; FUS2 group, 12.67 ± 1.12 s; Sham group, 7.33 ± 0.92 s; *p* < 0.01 (*p*=0.003) (Figures 2(d) and 2(e)). Conversely, there were no significant changes in the sciatic nerve index and body weight (Figures2(f) and 2(g)).

### 3.2. FUS Stimulation Promptly Improved the Mechanical Withdraw Threshold in the Long-Term Experiment

The results showed that the mechanical withdrawal threshold of the surgical side increased in the FUS group compared with that in the Sham group. This increase was sustained until the end of the FUS stimulation period (FUS group, 0.17 ± 0.04 g; Sham group, 0.06 ± 0.02 g; *p* < 0.05 (*p*=0.03)) (Figure 3(b)). There were no significant changes in the contralateral mechanical withdrawal threshold (*p*=0.19) (Figure 3(b)), bilateral thermal withdrawal threshold (surgical side *p*=0.21, other side *p*=0.25) (Figures 3(d) and 3(e)), body weight (*p*=0.93) (Figure 3(g)), and sciatic nerve index (*p*=0.49) (Figure 3(f)) between the FUS and the Sham groups.

### 3.3. Ultrasound-Induced Inhibition of Neuronal Discharges from the ACC Slices

Representative traces of spikes recorded during three phases in different channels randomly selected showed the ultrasound-induced changes using MEA recording (Figure 4(b)). The assessment of the 29 cells indicated that the spike frequencies in the ACC slices were effectively inhibited during ultrasound, which was sustained following ultrasound stimulation (prior to FUS2, 0.224 ± 0.0023 Hz; during FUS2, 0.145 ± 0.0218 Hz; following FUS2, 0.142 ± 0.0176 Hz, ^*∗∗∗*^*p* < 0.001and prior to during: *p* ≤ 0.001, prior to following: *p*=0.002), Student's paired *t*-test (Figure 4(c)).

### 3.4. FUS-Induced Differences in Protein Expression Indicated Alterations in Pathways in the ACC

**Figure 5:**
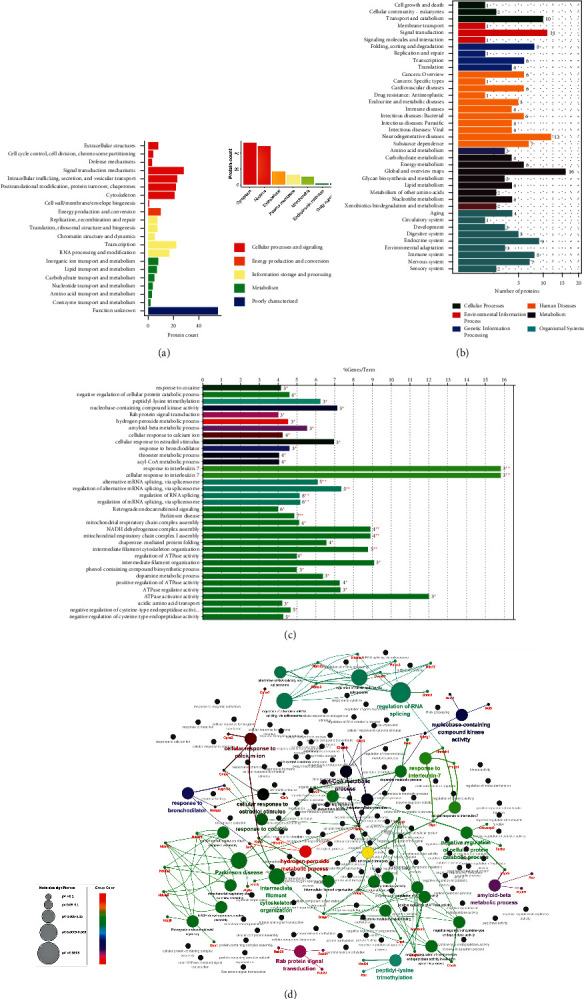
FUS-induced differences in protein expression indicated alterations in pathways in the ACC. EggNOG entry statistics (a). Subcellular location analysis (b). Visualized functional enrichment (c). Pathway analysis (d).

Protein profiling with a threshold of fold change of >1.2 and a *T*-test revealed differential up- and downregulations of 97 and 49 proteins, respectively; therefore, we observed a total of 146 protein expression changes. The subcellular structures of the proteins were mainly located in the cytoplasm, nucleus, and extracellular fluid (Figure 5(b)). Using EggNOG (EggNOG evolutionary genealogy of genes: Nonsupervised Orthologous Groups version 4.5.1, Computational Biology group-EMBL, Heidelberg), we found that the top 20 items (differential proteins) were involved in cellular processes, cellular signaling, and information storage and processing (Figure 5(a)). This suggests that ACC treatment with FUS alters cellular signal transduction and information processing involved in chronic pain. Further, enriched pathway analysis [32] (Cytoscape 3.7.2, Cytoscape Consortium) revealed that the differential proteins were mainly related to the endocrine, immune, and nervous systems (Figures 5(c) and 5(d)).

### 3.5. FUS2-Induced Changes in the Expression of Hnrnph1, Hnrnpd, Snrpb, and Dhx16

**Figure 6:**
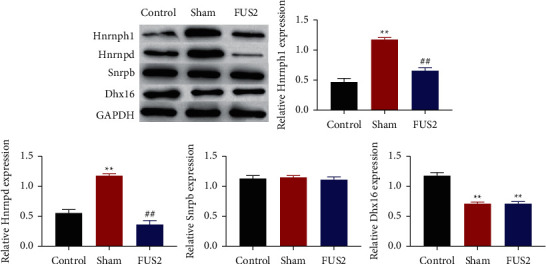
FUS2-induced changes in the expression of Hnrnph1, Hnrnpd, Snrpb, and Dhx16. The expression of Hnrnph1, Hnrnpd, Snrpb, and Dhx16 in tissue was measured by western blotting. ^*∗*^ indicates statistical significance between the Sham group and the Control group, the FUS2 group and the Control group. ^#^ indicates statistical significance between the FUS2 group and the Sham group.

Based on the plugin cytoHubba in Cytoscape, 4 key genes Hnrnph1, Hnrnpd, Snrpb, and Dhx16 were screened (Table S1). Western blotting results showed that HNRNPH1 and HNRNPD were significantly higher in the brain tissue of CCI mice compared with the normal group and the LUS stimulation group. Snrpb and Dhx16 expression shows no significant differences between CCI sham stimulation group and LUS stimulation group (Figure 6).

### 3.6. Safety and Temperature Evaluation

**Figure 7:**
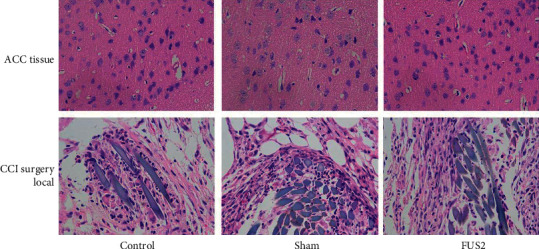
Safety and temperature evaluation. HE staining of the ACC brain tissue and the CCI surgery local site (×400).

The results of HE staining of ACC brain tissue and local parts of CCI operation in the Control group, Sham group, and FUS2 group showed that the sciatic nerve had no neurofibrillation, swelling, or thickening. Further, there were no axon irregularities, the disappearance of changes, or myelin loosening, disintegration, or precise segmental loss. There was no substantial change in the number of nerve cells in the brain, especially the ACC. Moreover, there was no inflammatory cell infiltration between the stroma and blood vessels, nerve cell swelling, ulceration, pyknosis, coagulative necrosis, vesicular degeneration, or vacuolar degeneration (Figure 7).

## 4. Discussion

Transcranial FUS is a popular technique used for noninvasive neuromodulation in both animal models and humans [20]. Our findings indicated that ultrasound stimulation of the ACC increased the mechanical withdraw threshold in the CCI mice model in both the short- and long-term experiments. In the short experiment, the mechanical threshold of the intervention group started increasing at 2 weeks following the ultrasound, and we observed a substantial difference in the mechanical threshold between the intervention group and the Sham group at 3 weeks. In the long-term experiment, there was an early and sustained increase in the threshold following ultrasound stimulation. Similar to related studies in recent years, protein analysis results of the ACC region showed that it mainly involves the immune system and endocrine system considering the occurrence of pain, especially the occurrence of chronic pain, which may involve changes in the functions of the CNS [33–37]. In addition, HNRNPH1 and HNRNPD may become important regulatory targets for FUS to stimulate reduce neuropathic pain.

Ultrasound stimulation is a noninvasive technique that allows accurate targeting of different neurological diseases. Additionally, it is a safe and cheap therapeutic method [38–41]. The ultrasound is even safer in cases where pacemakers or other important implantable devices are not antimagnetic or antielectric. Compared with TMS and tDCS, focused ultrasound is easier to implement in the deep brain area, making it safer and more accurate and improving the effects of uneven skull bones in humans or large animals through combination with magnetic resonance imaging (MRI) [42]. Similar to magnetic stimulation, FUS can be used in regions of the peripheral and central nervous systems [43]. Moreover, they both require more effective treatment following repeated stimulation for pain management. The difference between the protocols is that the cortical M1 is a more effective target for central inhibition using repetitive transcranial magnetic stimulation [16, 44]. At present, FUS is also widely used in larger animals. Low-intensity FUS modulated the excitability of regional brain tissues reversibly and safely in awake sheep [45]. In addition, FUS stimulation of female macaque monkeys under the guidance of MRI can double suppressive and excitative modulation of specific functional circuits [46]. FUS is different from other pharmacological or interventional therapies for acute and chronic pain. In our study, FUS stimulation of the ACC was effective in both the short- and long-term experiments, which suggests that FUS is a useful and noninvasive technique for pain management. Yang et al. reported increased neuronal excitability in ACC during nerve injury pain [47], especially in the bilateral ACC [48]. Similar to previous reports on other neuroregulatory methods (e.g., optogenetics) [13, 49–51], our MEA recordings revealed that FUS-induced reduction in the nerve excitability of the ACC in the brain slices was involved in pain attenuation, which also suggested that ACC could be an ideal target in CNS regulation of NP. Clennell et al. confirmed that there was a sustained effect until 8 hours after ultrasound stimulation [52], and we had found there was inhibition within 100s after ultrasound stimulation through MEA. In our experiment, the mechanical analgesic effect of ultrasound stimulation lasting for 3 weeks may be due to the continuous inhibition of nerve by repeated stimulation.

Peripheral nociceptor sensitization and durable synaptic plasticity in the CNS contribute to chronic pain in rodent models [53]. Although chronic and acute pain share common neural pathways [54, 55], establishing mechanisms for alleviating acute or chronic pain remains challenging. In a rodent NP model, central sensitization may be indicated by the characteristic potentiation of synaptic responses in the ACC and the development of allodynia at 1–2 weeks following nerve injury [56, 57]. This may explain the earlier presentation of FUS effects in the long-term experiment. Contrastingly, in the short-term experiment, the FUS-induced effects required a longer time (two weeks following the surgery) to appear.

The difference in the time of onset of FUS effects between the short- and long-term experiments indicated that the ACC or the early stage of the pain was not the optimal target for pain improvement in the short-term experiments. Conversely, central sensitization may have been the main target for pain regulation in long-term tests. Our findings indicate that FUS stimulation of the ACC has a therapeutic effect on chronic pain following peripheral nerve injury through changes in the nervous and immune systems of the CNS. Similarly, a previous study that focused on similar peripheral damage reported that the emergence of central sensitization played a vital role in the resulting chronic pain [58]. The regulation of different brain regions would have different results from the literature [59–67], and ultrasound regulation of ACC improved mechanical retraction threshold in the CCI model in our study, suggesting that ultrasound stimulation of different target brain regions can be considered to obtain better curative effect according to the different pain symptoms.

The occurrence and development of NP are caused by many factors and the disorder of genes regulation. Recent studies have found that FUS stimulation can improve the progress of NP by regulating the disordered genes [68]. In this study, we found four key genes Hnrnph1, Hnrnpd, Snrpb, and Dhx16 by analyzing the protein profile of long-term experimental mice. Further research found that Hnrnph1 and Hnrnpd are highly expressed in the brain tissue of NP mice, but downregulated in normal mouse brain tissue and FUS stimulation. HNRNPH1 and HNRNPD (heterogeneous nuclear ribonucleoprotein D) is a multifunctional RNA binding protein (RBP) with roles in regulation of alternative splicing, mRNA transcription, RNA stability, RNA localization, and regulation of target transcript translation. HNRNPH1 is abnormally overexpressed in a variety of tumors. Previous studies have confirmed that the high expression of HNRNPH1 can promote tumor development by inhibiting tumor suppressor genes [69]. However, the role of HNRNPH1 in NP has not been investigated. HNRNPD participates in the regulation of cell oxidative stress and inflammation. And it is also related to the cross-regulation of inflammation [70]. In addition, HNRNPD participates in the apoptosis of diabetic cells [71]. But the role of HNRNPD in NP has not been reported. According to the results of this experiment, we speculate that HNRNPH1 and HNRNPD may play a key role in NP, and, at the same time, FUS stimulation can directly or indirectly downregulate the levels of HNRNPH1 and HNRNPD to improve the development of NP. However, to determine the regulatory mechanism of HNRNPH1 and HNRNPD in NP, further research and investigation are needed.

Ultrasound waves have mechanical, cavitation, and thermal effects on biological tissue. The thermal effects of high-intensity FUS (HIFU >200 W/cm^2^) are reported to cause coagulative necrosis of brain tissue through the intact skull [72, 73]. Findings on HE staining and local temperature increase (Δ*T* < 0.1°C) showed that the treatment was safe and did not cause tissue damage to the target tissue and the surrounding. Moreover, the maximum negative peak pressure was much lower than the inertial cavitation threshold (40 MPa), which prevented tissue damage [74].

In conclusion, previous studies have indicated the safety of transcranial ultrasound stimulation, which is consistent with the results of other studies [21, 75, 76]. Our findings indicate that FUS effectively alleviates mechanical NP via ACC inhibition, especially in the chronic state. The underlying mechanisms may be associated with several central sensitization stages, suggesting that different protocols may be more appropriate for different NP stages. Protein analysis in the long-term experiment demonstrated that FUS-induced neuromodulation of the ACC altered the immune function and several pathways involved in central sensitization. These results suggested great potential for clinical translation. Particularly, it is of significance to select different intervention programs at different clinical stages. However, ultrasound is prone to be off-target due to the heterogeneity of the skull, and MRI is needed to correct the ultrasound stimulation in humans or large animals. Based on ethical principles, we could not use a larger sample of mice to verify every parameter, and there may be more reasonable parameters inducing different effects. Future studies should investigate targeted brain regions, appropriate time points, and response pathways to identify the mechanisms underlying FUS treatment of NP.

## Data Availability

The data used to support the findings of this study are available from the corresponding author upon reasonable request.

## Supplementary Materials

Supplementary Materials Table S1: cytoHubba function results.
