# Supplementary material for: Transcranial Ultrasound Stimulation of the Anterior Cingulate Cortex Reduces Neuropathic Pain in Mice
Source: Evid Based Complement Alternat Med. 2021 Dec 31;2021:6510383. doi: 10.1155/2021/6510383 (PMC8741380; doi:10.1155/2021/6510383)
Supplement: Supplementary Materials — Table S1: cytoHubba function results. [file 6510383.f1.pdf]

Table S1 cytoHubba function results

node_name	MCC	MNC	Degree	BottleNeck	EcCentricity	Closeness	ClusteringCoefficient
Hnrnph1	5189	13	14	7	0.07037	23.67143	0.37363
Hnrnpd	5167	9	10	4	0.07037	21.17143	0.6
Snrpb	5178	10	10	1	0.07037	21.42143	0.66667
Dhx16	5053	9	10	5	0.07037	21.25476	0.55556
Cpsf2	5047	8	9	57	0.07917	21.75119	0.63889
Xab2	5166	9	9	3	0.07037	20.50476	0.75
Khdrbs1	23	7	8	2	0.06333	19.08889	0.32143
Snrnp40	5160	8	8	1	0.07037	20.00476	0.89286
H2afz	8	3	7	9	0.06333	15.84087	0.09524
Aqr	5040	7	7	1	0.07037	19.50476	1
Ddx17	19	5	6	3	0.06333	18.42222	0.46667
Gnb1	6	1	6	57	0.09048	19.6119	0
Hnrnpab	120	5	5	1	0.06333	17.33889	1
Atp5e	30	5	5	1	0.03056	6.66667	0.8
Ndufa2	30	5	5	2	0.03056	6.66667	0.8
Ndufa8	25	4	5	11	0.04074	7.16667	0.6
Ndufs5	30	5	5	1	0.03056	6.66667	0.8
Cryab	4	2	4	3	0.07917	15.1619	0.33333
Sod1	5	3	4	12	0.09048	17.47143	0.33333
Snca	5	3	4	1	0.07917	16.41429	0.33333
Rbfox3	4	2	4	7	0.07917	15.62857	0.33333
Ndufs4	24	4	4	1	0.03056	6.16667	1
Dnaja1	4	3	3	1	0.07037	13.97817	0.66667
Ppil2	6	3	3	1	0.07037	16.2881	1
Ewsr1	6	3	3	1	0.06333	16.42222	1

Dnajb6	4	3	3	2	0.07037	13.97817	0.66667
Dnajb4	3	2	3	4	0.07917	15.5131	0.33333
Timm13	6	3	3	1	0.02444	5.23333	1
Nme1	3	1	3	57	0.10556	18.78333	0
Ppp1r1b	3	2	3	2	0.07037	13.18254	0.33333
Atrx	4	3	3	1	0.05758	12.25804	0.66667
Pvalb	3	2	3	4	0.07037	13.43254	0.33333
Suclg1	3	1	3	11	0.04074	6.33333	0
Sec61a1	3	1	3	4	0.04444	3	0
Krt10	3	2	3	2	0.04444	3	0.33333
Chp1	2	1	2	2	0.05758	9.85848	0
Rbm14	2	2	2	1	0.06333	15.67222	1
Ace	2	1	2	3	0.03333	2	0
Sncb	2	1	2	3	0.03333	2	0
Hmgb1	2	1	2	2	0.05758	11.92471	0
Prkar1a	2	1	2	4	0.07917	14.97976	0
Capn7	2	1	2	3	0.03333	2	0
